# A45 EXPLORING COST AND VALIDITY OF NOVEL COMMERCIAL AND NON-COMMERCIAL (SELF-ASSEMBLED) SIMULATORS IN GASTROINTESTINAL ENDOSCOPY TRAINING: A SCOPING REVIEW

**DOI:** 10.1093/jcag/gwae059.045

**Published:** 2025-02-10

**Authors:** A Y Zhao, N Gimpaya, J Lisondra, A Wiggin, A Almneni, W Tran, S Grover

**Affiliations:** Scarborough Health Network, Scarborough, ON, Canada; Scarborough Health Network, Scarborough, ON, Canada; Education Research, Scarborough Health Network, Scarborough, ON, Canada; Scarborough Health Network, Scarborough, ON, Canada; Scarborough Health Network, Scarborough, ON, Canada; Education Research, Scarborough Health Network, Scarborough, ON, Canada; Scarborough Health Network, Scarborough, ON, Canada

## Abstract

**Background:**

Simulation-based training improves trainee learning curves and minimizes patient risk. However, not all endoscopic procedures have commercially available simulation models. Further, costs and location may limit trainee access to commercial simulators. This has driven the design and development of non-commercial, self-assembled simulators by educators, training programs, and learners.

**Aims:**

To perform a scoping review to explore reporting of validity, assembly time and cost of novel simulators developed in the last 5 years.

**Methods:**

A search on MEDLINE, PubMed, and ProQuest from January 2019 to September 2024 identified articles that developed or used simulators for gastrointestinal (GI) endoscopy procedures and techniques. A five-year date limit was implemented to account for advancement in simulation technology over this time frame. Two independent reviewers screened the articles. Included articles were designated as non-commercial simulators if they are open-access, not for purchase, and intended for self-assembly; or commercial simulators if they did not meet these points. Data on cost, assembly time, and method of validity testing were recorded. Costs were converted to USD using a consumer price index calculator (2024). The primary outcome was the type of validity testing as reported in the articles. Other outcomes are the differences between cost and assembly time.

**Results:**

28 articles were included. Figure 1 summarizes each study, the characteristics and the specific skills simulated for each simulator, and the methods used for validity testing. 19 articles were on non-commercial simulators (18 mechanical, 1 Virtual Reality (VR)) and 9 were on commercial simulators (7 mechanical and 2 VR). Validity testing was conducted in 12 of the 19 non-commercial simulator studies (9 instances of face validity, 10 content, and 11 construct), and in 8 of the 9 commercial simulator articles (3 instances of face validity, 2 content, and 2 construct). The single study on a non-commercial VR simulator did not report cost. Costs ranged from $28-$1035, for non-commercial mechanical simulators, $247-$12,995 for commercial mechanical simulators, and $62,087-$136,591 for commercial VR simulators. Only four articles on mechanical simulators reported assembly time, which ranged from 1-8 hours, with additional set-up ranges of 10-20 minutes.

**Conclusions:**

This scoping review explored the validity testing, cost, preparation time and types of validity used in newly-developed commercial and non-commercial simulators. Future iterations of this study will expand the search strategy, assess the quality of studies, evaluate and compare the validity testing performed between commercial and non-commercial simulators.

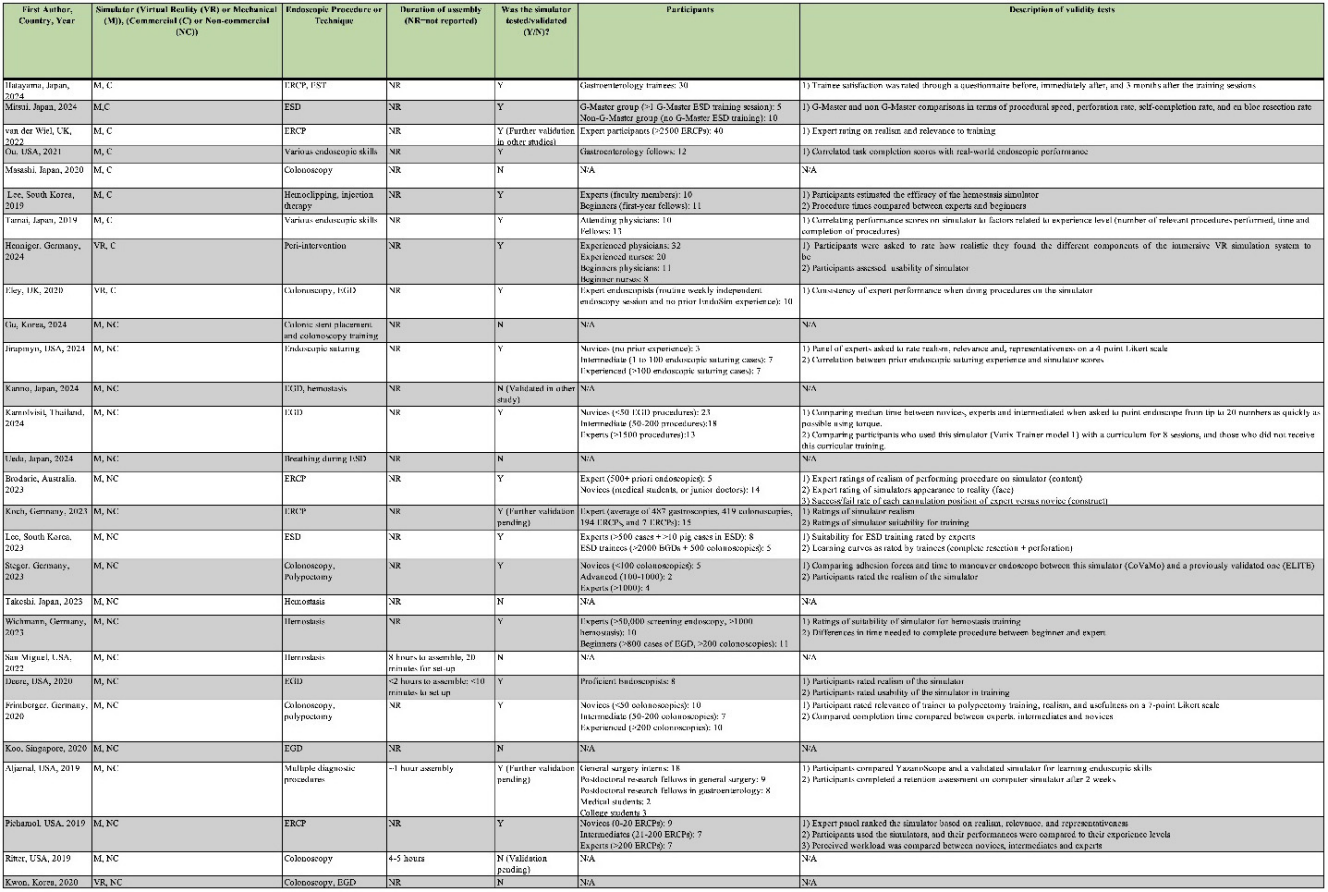

Included articles.

**Funding Agencies:**

None

